# *Notes from the Field:* A Cluster of Multi-Strain Invasive Pneumococcal Disease Among Persons Experiencing Homelessness and Use of Pneumococcal Conjugate Vaccine — El Paso County, Colorado, 2022

**DOI:** 10.15585/mmwr.mm7246a5

**Published:** 2023-11-17

**Authors:** Jessica Callaway, Kristi Durbin, Haley Zachary, Meghan M. Barnes, Miwako Kobayashi, Sopio Chochua, Natalia Gayou, Bernadette Albanese

**Affiliations:** ^1^El Paso County Public Health, Colorado Springs, Colorado; ^2^Colorado Department of Public Health & Environment; ^3^Division of Bacterial Diseases, National Center for Immunization and Respiratory Diseases, CDC.

Persons experiencing homelessness are often at increased risk for invasive pneumococcal disease (IPD)* due to underlying health conditions or risk factors (risk conditions) (*1*,*2*). Homelessness alone is not an indication for pneumococcal vaccination according to current Advisory Committee on Immunization Practices (ACIP) recommendations (*3*): adults aged ≥65 years or 19–64 years with certain underlying medical conditions or risk factors^†^ with no previous or unknown history of receipt of pneumococcal conjugate vaccine (PCV) should receive 1 dose of either 20-valent or 15-valent PCV (PCV20 or PCV15, respectively). On November 29, 2022, El Paso (Colorado) County Public Health (EPCPH) was informed by a single hospital of three cases of IPD among persons experiencing homelessness, with all illness onset dates occurring within a single week.

## Investigation and Outcomes

EPCPH initiated active surveillance at all local hospitals to identify additional IPD cases in persons experiencing homelessness. A case was defined as a diagnosis of IPD in a person aged ≥18 years experiencing homelessness in El Paso County, Colorado, during November 1, 2022–January 28, 2023. Analysis of cases was conducted to describe demographic characteristics, clinical presentation, codetection of respiratory viruses (based on testing requested by the treating physician), underlying medical conditions, and shelter use. Pneumococcal isolates from patients with IPD were serotyped at CDC by Quellung reaction^§^ and whole genome sequencing. The activity was reviewed by CDC, deemed not research, and was conducted consistent with applicable federal law and CDC policy.^¶^

Twelve persons experiencing homelessness with IPD were identified, six of whom used housing and social services at the same local shelter serving persons experiencing homelessness. Nine of the 12 patients were male, and eight were aged <50 years. All had bacteremia, and nine also received a diagnosis of pneumonia. Ten patients were hospitalized for a median of 9 days (range = 3–14 days); no deaths were reported. Viral coinfections were identified in four patients, including both SARS-CoV-2 and rhinovirus (one patient), respiratory syncytial virus (one), human metapneumovirus (one), and SARS-CoV-2 (one). Underlying health conditions or risk factors included substance abuse (nine patients), current smoking (five), alcoholism (three), and diabetes (one). Seven of 10 pneumococcal isolates with serotyping results were serotype 4. Whole genome sequencing and single nucleotide polymorphism (SNP) analysis of serotype 4 isolates showed that most isolates were not genomically related.^**^ Other serotypes identified were serotype 8 (one), 9N (one), and 19F (one); 90% of serotyped isolates (all except 9N) are contained in PCV20.

Fifteen days after receiving the initial report of IPD cases, EPCPH initiated the first of five vaccination clinics at three local facilities serving persons experiencing homelessness (Figure). To avoid delays in administering the vaccine, clinics were held before the serotyping results were available. A total of 87 PCV20 doses were administered.

**FIGURE:**
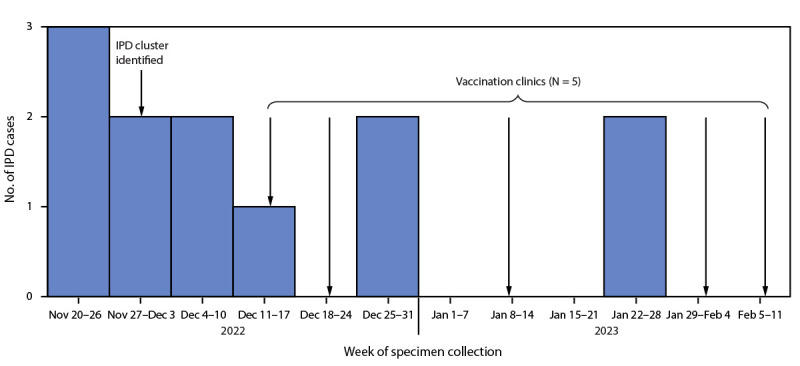
Weekly number of cases of invasive pneumococcal disease among persons experiencing homelessness and the number of El Paso County Public Health vaccination clinics — El Paso County, Colorado, November 2022–February 2023 **Abbreviation:** IPD = invasive pneumococcal disease.

## Preliminary Conclusions and Actions

Rapid implementation of targeted vaccination clinics for any person aged ≥18 years experiencing homelessness facilitated the efficient delivery of vaccine and served to expand reach to this population. No new IPD cases were reported among persons experiencing homelessness during January 29, 2023–April 15, 2023; however, during this period, one unvaccinated person experiencing homelessness from the cluster with multiple IPD risk conditions experienced recurrent IPD. Since that recurrent infection, one additional case was reported, during the week of August 23, 2023.

IPD among persons experiencing homelessness remains a public health concern. Crowding, substance abuse, chronic health conditions, and lack of consistent health care and access to routine vaccination services place persons experiencing homelessness at increased risk for pneumococcal disease (*1*,*2*,*4*). In addition, pneumococcal vaccination coverage among younger U.S. adults who are recommended to receive the vaccine based on risk conditions (*3*) has been inconsistent and low (*5*). In this epidemiologic cluster, most pneumococcal serotypes identified were contained in PCV20, and vaccine was administered to prevent additional IPD cases. Pneumococcal vaccination of persons experiencing homelessness should be considered standard health care if they have risk conditions for which ACIP recommends PCV use (*3*). 

## Footnotes

* Isolation of or identification by polymerase chain reaction of *Streptococcus pneumoniae* from a normally sterile body site.

† Underlying medical conditions or risk factors include alcoholism; chronic heart, liver, or lung disease; chronic renal failure; cigarette smoking; cochlear implant; asplenia; cerebrospinal fluid leak; diabetes mellitus; malignancy; HIV; Hodgkin disease; immunodeficiency; iatrogenic immunosuppression; hematopoietic neoplasms; nephrotic syndrome; solid organ transplant; sickle cell disease; and hemoglobinopathies.

§ A method for pneumococcal capsular serotyping involving testing of a pneumococcal cell suspension with specific antisera and microscopic observation of the antigen-antibody reaction.

¶ 45 C.F.R. part 46, 21 C.F.R. part 56; 42 U.S.C. Sect. 241(d); 5 U.S.C. Sect. 552a; 44 U.S.C. Sect. 3501 et seq.

** The average pairwise SNP distance between serotype 4 isolates was 89 SNPs. The minimum SNP distance was seven SNPs between one pair of isolates, and the maximum SNP distance was 252 SNPs. For isolates to be considered genomically clustered, they should be highly related to each other, generally different by 10 or fewer SNPs. Only two out of seven serotype 4 isolates were highly genomically related.
